# Genome-Wide Identification and Functional Investigation of *1-Aminocyclopropane-1-carboxylic Acid Oxidase* (*ACO*) Genes in Cotton

**DOI:** 10.3390/plants10081699

**Published:** 2021-08-18

**Authors:** Hengling Wei, Yujun Xue, Pengyun Chen, Pengbo Hao, Fei Wei, Lu Sun, Yonglin Yang

**Affiliations:** 1 State Key Laboratory of Cotton Biology, Institute of Cotton Research of CAAS, Anyang 455000, China; henglingwei@163.com (H.W.); Xyujun2021@163.com (Y.X.); arpengyun@163.com (P.C.); hao_pengbo@163.com (P.H.); weifei888000@outlook.com (F.W.); 2 Handan Academy of Agricultural Sciences, Handan 056001, China; sunlu1217.ok@163.com; 3 Shihezi Academy of Agricultural Sciences, Shihezi 832099, China

**Keywords:** *G. hirsutum*, ACO gene, expression patterns, flowering, stress response

## Abstract

ACO is one of the rate-limiting enzymes in the biosynthesis of ethylene, and it plays a critical role in the regulation of plant growth and development. However, the function of *ACO* genes in cotton is not well studied. In this study, a total of 332 *GhACOs*, 187 *GaACOs*, and *181 GrACOs* were identified in *G. hirsutum*, *G. arboretum*, and *G. raimondii*, respectively. Gene duplication analysis showed that whole-genome duplication (WGD) and tandem duplication were the major forces driving the generation of cotton *ACO* genes. In the promoters of *GhACOs*, there were cis-acting elements responding to stress, phytohormones, light, and circadian factors, indicating the possible involvement of *GhACOs* in these processes. Expression and co-expression analyses illustrated that most *GhACOs* were not only widely expressed in various tissues but also coexpressed with other genes in response to salt and drought stress. *GhACO106_At* overexpression in *Arabidopsis* promoted flowering and increased salt tolerance. These results provide a comprehensive overview of the *ACO* genes of cotton and lay the foundation for subsequent functional studies of these genes.

## 1. Introduction

Ethylene has a variety of physiological regulatory functions throughout the life of plants, including roles in flower formation, fruit maturation, and senescence [1,2,3,4]. ACO is the last enzyme in the ethylene synthesis pathway [1,3]. The first identified ACO gene was *PTOM13*, which was cloned from tomato fruit in 1985 and confirmed as an ethylene-forming enzyme in 1991 [5].

In tobacco, inhibiting the expression of an *ACO*-like gene leads to female sterility of transgenic plants and this functional defect can be recovered by treatment with ethylene [6]. In cucumber, *CsACO2* was found to be the main gene required for the development of carpels [7]. In tomato, with the exception of *LeACO2*, all the other five *ACO* genes are expressed during different stages of fruit ripening, and *ACO* transgenic tomato fruits are more resistant to storage and shrinkage than control fruits, showing that *ACO* genes are related to fruit ripening in tomato [8,9,10,11,12]. In apple, peach, strawberry, and banana, the expression of *ACO* genes also changes during fruit ripening, so it is hypothesized that the *ACO* genes have a universal function related to fruit ripening [13,14,15,16]. In carnation, *ACO* gene expression can be induced during flowering, pollination, and petal senescence [17,18]. In soybean, Zabala et al. hypothesized that the overexpression of an *ACO* gene precedes the development of minute hilum seed coat phenotype [19].

The *ACO* genes have also been reported to be involved in the responses to environmental stress and hormones [20,21]. In *Arabidopsis*, *TaACO1* can be negatively regulated by salinity stress [22]. Additionally, flood tolerance develops following the overexpression of an *ACO* gene in *Arabidopsis* [23]. In sunflower, under salt stress, nitric oxide was found to regulate lateral root formation through the modulation of *ACO* gene activity [24]. In cucumber and tomato, *ACO* gene expression can be induced by wounding [10,25]. In potato, the mRNA level of *ST-ACO3* increases following inoculation with *F. eumartii* [26]. In tomato and cauliflower, *ACO* genes have been found to respond to ABA and mechanical damage [27,28]. The expression of *OsACO1* can also be induced by external mechanical damage, and IAA can significantly increase the expression of *OsACO2* while completely inhibiting that of *OsACO3* [29,30]. In *Agrostis stolonifera*, *AsACO* is strongly upregulated in response to ethephon (ETH), methyl jasmonate (MeJA), salicylic acid (SA), and cold temperature but is downregulated in response to drought and salt stresses [31].

*Gossypium* originated approximately 10 million years ago and then experienced rapid speciation and differentiation events [32]. To date, 52 cotton genera, including 45 diploid and 7 natural tetraploid genomes, have been discovered and named [33]. It has been theorized that allotetraploid upland cotton (AADD, 2n = 4× = 52) probably originated approximately 1–2 million years ago (MYA) via the natural hybridization of the diploid genomes of *G. arboreum* (AA, 2n = 2× = 26) and *G. raimondii* (DD, 2n = 2× = 26) [34]. As the main generator of natural fibers for the textile industry, the genus *Gossypium* is widely planted worldwide. The planting area of upland cotton (*G. hirsutum*) accounts for more than 95% of the global cotton planting area due to its excellent agricultural traits, such as high yield, superior fiber, and strong stress tolerance. In addition, the members of the *Gossypium* genus are used as model plants for studying plant cell elongation, cell wall and cellulose synthesis, and polyploid evolution.

As a multigene family with multiple functions, *ACO* genes have been cloned from a variety of plants such as melon, carnation, and *Arabidopsis* [17,20,35,36,37]. However, the identification and functional characterization of *ACO* genes in cotton is rarely reported. In this study, *ACO* genes were identified from three cotton species, and their physicochemical properties, domain architectures, phylogenetic relationships, syntenic relationships, and spatiotemporal expression were analyzed. Furthermore, the function of an *ACO* gene, *GhACO106_At*, was investigated by performing an overexpression assay in *Arabidopsis*.

## 2. Results

### 2.1. Identification and Sequence Analysis of ACO Genes in Cotton

In total, 332 *GhACOs*, 187 *GaACOs*, and 181 *GrACOs* were identified from *G. hirsutum* (*Gh*), *G. arboretum* (*Ga*), and *G. raimondii* (*Gr*), respectively. Basic information on these *ACO* genes is listed in Supplementary Table S1. The lengths of the proteins encoded by these *ACO* genes ranged from 191 to 979 amino acids (aa); their molecular weights (MWs) ranged from 21,926.19 to 110,977.16; their pI values ranged from 4.64 to 9.72; and their grand average of hydropathicity (GRAVY) values ranged from −0.607 to 0.01 (−0.021). These *ACO* genes were divided into four subgroups based on phylogenetic analysis. The largest group (Group IV) contained 277 genes, the smallest group (Group I) contained only 63 genes, and Group II and Group III each contained 180 genes (Supplementary Figure S1). In total, 10 conserved motifs were detected in these cotton ACO proteins. Among these 10 motifs, motif 9 was the most conserved domain and was found in 698 cotton ACO proteins (Figure S2 and Table S2).

**Figure 1:**
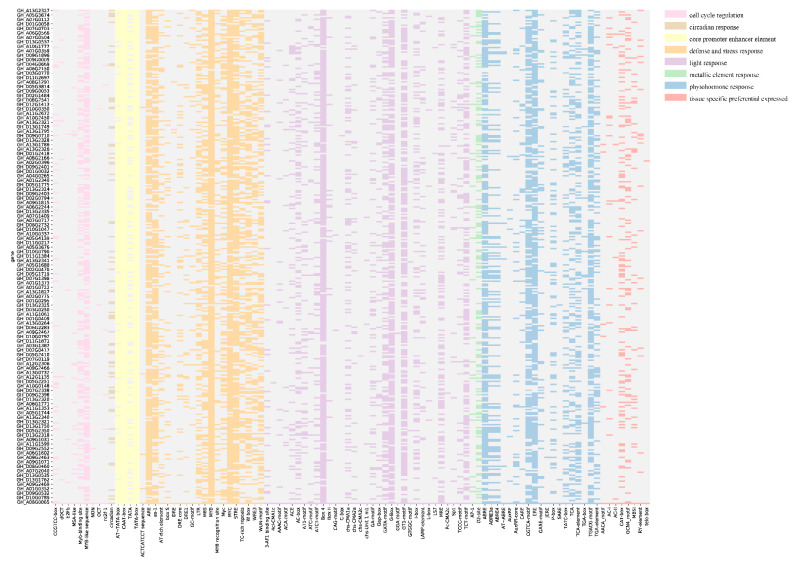
Cis-elements in the promoters of *GhACOs*. The cis-elements with similar functions are shown in the same blocks with the same color.

The upstream sequences of the *GhACOs* were retrieved, and cis-acting elements were predicted. The results showed that the promoters included elements that respond to stress, phytohormones, and light. The stress response elements included drought response (DRE core, DRE1, MBS, MYB recognition site, MYC, and as-1), water stress response (MYB and AT-rich element), heat stress response (STRE), metal response (AP-1 and O2-site), wounding and pathogen response (W box, box S, WRE3, and WUN-motif), and anoxic response (GC-motif) elements. The phytohormone response elements included elements that respond to ETH (ERE), ABA (ABRE, ABRE2, ABRE3a, ABRE4, and CARE), IAA (AuxRE and TGA-element), gibberellins (GARE-motif, P-box, and TATC-box), jasmonic acid (CGTCA-motif and TGACG-motif), and salicylic acid (TCA-element). The light response elements included the ACA-motif, GT1-motif, TCT-motif, chs-CMA1a, and Box 4. Other elements, such as lycopersicon esculentum cis-acting regulatory element (CAAAGATATC), which is involved in circadian control and cell cycle regulation, were also predicted. Detailed information on the cis-elements is shown and listed in Figure 1 and Table S3.

### 2.2. Duplication Events and Selection Pressure Analysis of ACO Genes in Cotton

**Figure 2:**
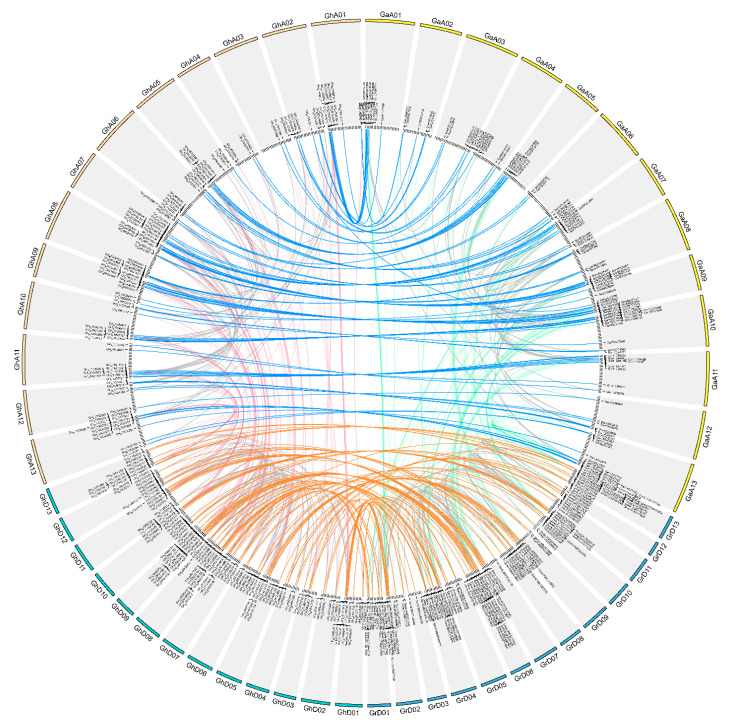
Collinear *ACO* gene pairs in cotton. Yellow, blue, orange, and green represent the chromosomes of *Ga*, *Gr*, and the At and Dt subgenomes of *Gh*, respectively. Gray lines represent intragenomic gene pairs, while other colors represent intergenomic gene pairs.

To study the replication and expansion mechanisms of *ACO* genes in cotton, data on intragenomic duplications were analyzed. A total of 101, 96, 100, and 98 gene pairs were identified as intragenomic duplications in *Ga*, *Gr*, the At subgenome of *Gh*, and the Dt subgenome of *Gh*, respectively (Figure 2 and Table S4). Among the duplication events, WGD (51.90%) and tandem duplication (24.30%) were the major drivers of the formation of these cotton *ACO* genes. The nonsynonymous substitution rate (Ka)/synonymous substitution rate (Ks; Ka/Ks) values of the duplicated gene pairs in *Ga*, *Gr*, the At subgenome of *Gh*, and the Dt subgenome of *Gh* ranged from 0.057 to 1.106 (average 0.254), 0 to 0.818 (average 0.219), 0 to 0.959 (average 0.225), and 0.056 to 0.855 (average 0.228), respectively.

Intergenomic duplication events between *Ga* and *Gr*; *Ga* and the At subgenome of *Gh*; *Gr* and the Dt subgenome of *Gh*; and the At and Dt subgenomes of *Gh* were detected using MCScanX. The numbers of intergenomic duplication gene pairs identified were 112, 108, 119, and 110, respectively (Figure 2 and Table S4). The Ka/Ks values of the intergenomic pairs between *Ga* and *Gr* ranged from 0.061 to 1.058 (average 0.322); those between *Ga* and the At subgenome of *Gh* ranged from 0.054 to 1.920 (average 0.462); those between *Gr* and the Dt subgenome of *Gh* ranged from 0.068 to 1.958 (average 0.428); and those between the At and Dt subgenomes of *Gh* ranged from 0.050 to 0.977 (average 0.329).

### 2.3. Expression and Co-Expression Patterns of GhACOs

**Figure 3:**
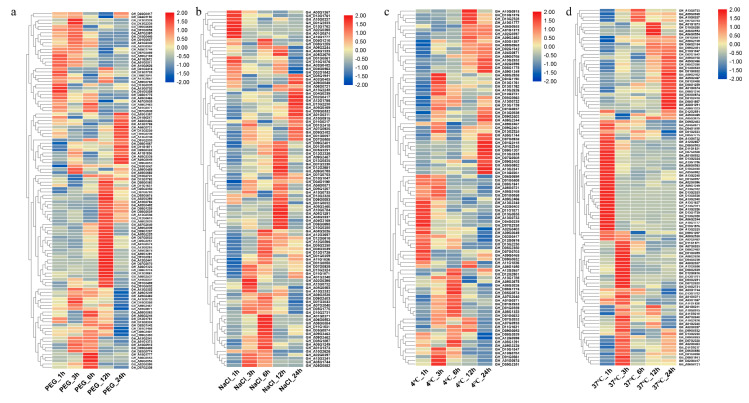
Expression patterns of *GhACOs* under drought, salt, cold, and heat stresses, (**a**): drought stress condition, (**b**): salt stress condition, (**c**): cold stress condition, (**d**): heat stress condition.

Using the available transcriptome data, the expression characteristics of *GhACOs* in different tissues were determined. Thirty-five *GhACOs*, such as *GhACO180_At* (*GH_A06G1771*) and *GhACO181_D*t (*GH_D05G1775*), were widely expressed in various tissues, including anther, bract, filament, leaf, petal, pistil, root, sepal, stem, and torus tissues as well as in ovules and fibers at different developmental stages (Figure S3 and Table S5). Nineteen genes, such as *GhACO275_At* (*GH_A02G1448*) and *GhACO269_Dt* (*GH_D09G0980*), showed no detectable expression in any tissue (Figure S3 and Table S5). Other genes expressed in only some of these tissues, such as *GhACO112_Dt* (*GH_D09G1767*), presented high expression in anthers, bracts, filaments, petals, pistils, roots, sepals, and toruses but no expression in ovules, fibers, leaves, or stems (Figure S3 and Table S5). The expression levels of *GhACOs* were also explored under drought (PEG 6000), salt (NaCl, 400 mmol·L^−1^), cold (4 °C), and hot (37 °C) stresses. According to a standard mean expression level (FPKM) greater than 1, it was found that 110, 101, 95, and 106 *GhACOs* with different expression patterns were involved in the responses to drought, salt, cold, and hot stresses, respectively (Figure 3 and Table S6).

**Figure 4:**
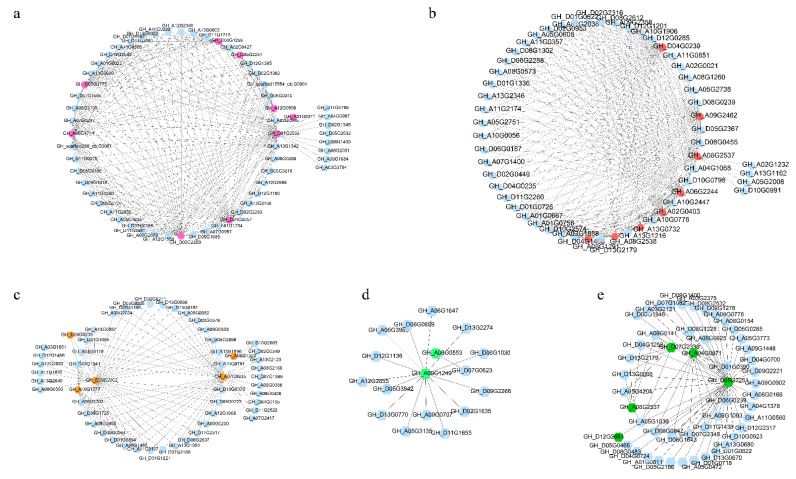
Co-expression networks of *GhACOs*. (**a**–**e**) Representative modules. Pink, red, orange, and green circles are *GhACOs*, and blue circles are the genes co-expressed with *GhACOs*.

Through co-expression analysis, we found that genes such as *GhACO110_Dt* (*GH_D01G2533*), *GhACO52_At* (*GH_A08G2537*), *GhACO187_At* (*GH_A07G0935*), *GhACO47_At* (*GH_A09G1249*), and *GhACO21_Dt* (*GH_D05G2251*) could be co-expressed with 227 genes in different modules (Figure 4 and Table S7). A large number of genes belonging to the major intrinsic protein (MIP) superfamily, which consists of membrane channels that selectively transport water, small neutral molecules, and ions transferred between cells, were discovered to be co-expressed with *GhACOs* (Figure 4 and Table S7). RRM_SF superfamily genes, also known as RNA binding domain (RBD) or ribonucleoprotein domain (RNP), were also found to be co-expressed with *GhACOs* (Figure 4 and Table S7). Genes belonging to WRKY transcription factor families (*GH_D07G1505*) that were related to salt stress or antifungal activities (*GH_D02G1545* and *GH_D06G1400*) were also shown to be co-expressed with *GhACOs* (Figure 4 and Table S7).

### 2.4. Function Determination of GhACO106_At on Flowering Time

**Figure 5:**
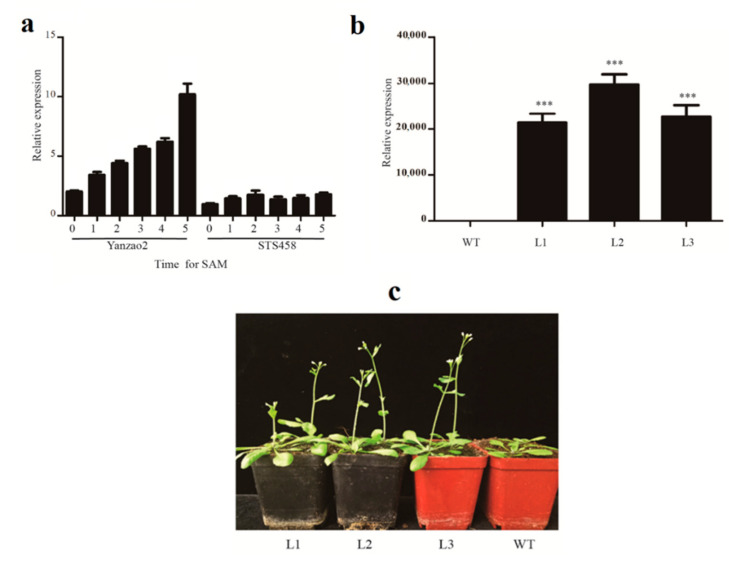
The expression of *GhACO106_At* increased with the development process of flower bud differentiation in the early-maturing variety (Yanzao2), and the overexpression of *GhACO106_At* in *Arabidopsis* caused early flowering. (**a**) *GhACO106_At* expression levels in cotton varieties with different maturation phenotypes and different shoot apical meristem (SAM) stages. Labels 0, 1, 2, 3, 4, and 5 indicate shoot apices in the two-cotyledon expanded stage and the first, second, third, fourth, and fifth expanded true leaf stages, respectively. (**b**) Verification of *Arabidopsis* lines positive for *GhACO106_At* transfer via PCR. WT: wild type; L1–L3: transgenic lines; Student’s *t*-test: *** *p* < 0.001. (**c**) Phenotypes of transgenic and WT *Arabidopsis* lines.

By analyzing the transcriptome data of upland cotton at different stages of maturation [38], we found that *GhACO106_At* (*GH_A09G0769)* showed clear differences in expression in early-maturing and late-maturing cotton varieties. In this research, the expression characteristics of *GhACO106_At* were further confirmed by qRT-PCR. The qRT-PCR results showed that the expression level of *GhACO106_At* in the early-maturing variety (Yanzao2) was higher than that in the late-maturing variety (STS458) at the same developmental stages. Furthermore, the expression level of *GhACO106_At* increased with the development of flower bud differentiation in Yanzao2 but showed no significant change in STS458 (Figure 5a).

To further investigate the function of *GhACO106_At* in cotton maturation, the *GhACO106_At* overexpression assay was performed in *Arabidopsis,* and T_3_ transgenic plants were used for phenotypic analysis (Figure 5b). Compared with the wild type (WT), all the independent transgenic lines showed relatively early flowering, and their numbers of rosette leaves were significantly reduced (Figure 5c and Table 1). This result indicated that *GhACO106_At* may play an important role in cotton maturity.

### 2.5. Function Analysis of GhACO106_At under Salt Stress

**Figure 6:**
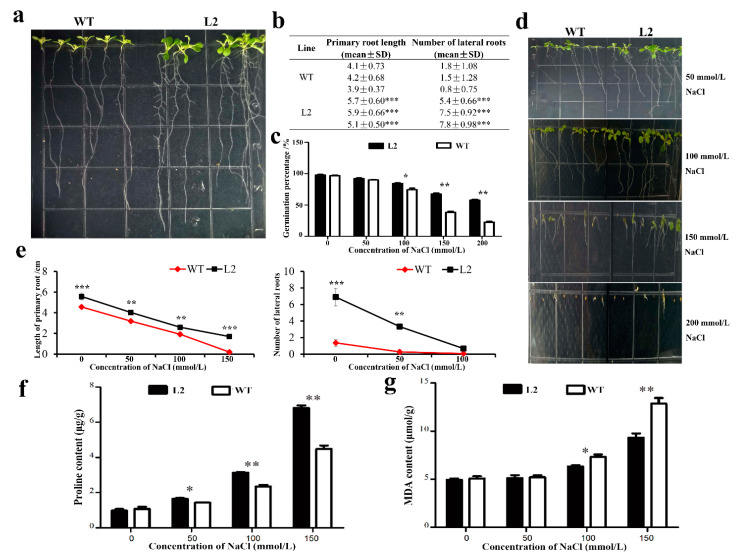
*GhACO106_At* increased salt stress tolerance in transgenic *Arabidopsis*. (**a**) Root phenotype of 8-day-old WT and L2 transgenic seedlings; (**b**) statistical data of primary roots and lateral roots of WT and L2 transgenic seedlings; (**c**) seed germination rate of WT and L2 transgenic seedlings under control and various NaCl treatments; (**d**) root phenotypes of 8-day-old WT and L2 transgenic seedlings under various NaCl treatments; (**e**) primary root lengths and the number of lateral roots of WT and L2 transgenic seedlings under control and various NaCl treatments; (**f**) proline contents of WT and L2 transgenic seedlings under control and various NaCl treatments; (**g**) MDA contents of WT and L2 transgenic seedlings under control and various NaCl treatments. L2: transgenic line, WT: wild type. Student’s *t*-test: * *p* < 0.05; ** *p* < 0.01; *** *p* < 0.001.

To further understand the function of *GhACO106_At*, transgenic *Arabidopsis* line L2, which showed the highest *GhACO106_At* expression level, was selected for a more detailed analysis. The L2 transgenic and WT seedlings were grown in 1/2 MS medium for 8 days. Compared to the WT, the L2 seedlings exhibited significantly longer primary roots and many more lateral roots (Figure 6a,b). These results suggested a positive role of *GhACO106_At* in plant root development and therefore a presumed role of this gene in mediating stress tolerance. We then investigated the potential effects of this gene by performing salt treatment. First, the germination of L2 and WT seeds was evaluated in media with various NaCl concentrations. In normal and 50 mmol·L^−1^ NaCl media, no obvious effect or difference was observed between transgenic *Arabidopsis* and the WT. On treatment with NaCl concentrations of 100 mmol·L^−1^ or higher, the transgenic plants showed significantly higher germination percentages than the WT plants (*p* < 0.05; Figure 6c). Furthermore, the transgenic *Arabidopsis* plants had longer roots and more lateral roots than WT *Arabidopsis* plants on all tested conditioned media (Figure 6d,e). These results illustrated the positive role of GhACO106_At in seed germination and root development. To precisely explore the effects of *GhACO106_At* under stress, the proline and MDA contents of 8-day-old transgenic *Arabidopsis* L2 line and WT plants were then investigated. Proline was induced by NaCl in both *GhACO106_At* and WT seedlings (Figure 6f). Moreover, as the salt concentration increased, the change in proline content in L2 was more significant than that in WT (Figure 6f). The MDA content was not obviously different between transgenic *Arabidopsis* and WT plants in normal and 50 mmol·L^−1^ NaCl media (Figure 6g). Furthermore, the MDA content of transgenic *Arabidopsis* plants was significantly lower than that of WT plants in media with 100 and 150 mmol·L^−1^ NaCl (Figure 6g). These results demonstrate that the overexpression of *GhACO106_At* can significantly enhance the resistance of plants to salt stress.

## 3. Discussion

### 3.1. Gene Duplication Events and Positive Selection Have Led to Large Numbers and Different Functions of ACO Genes in Cotton

Due to their increased amount of genetic material, polyploid plants show higher degrees of heterozygosity and genetic diversity as well as greater evolutionary adaptability [39]. For example, natural tetraploid *Arabidopsis thaliana* shows increased potassium uptake and salinity tolerance [40], and the polyploidization of rice and citrus alters the expression of hormone-related genes, making the plants more resistant to saline-alkali conditions and drought [41]. The *Gossypium* genus has experienced rapid speciation and differentiation events, as well as hybridization and polyploidization events. Allotetraploid upland cotton, which has excellent agricultural traits, was the outcome of such natural hybridization. Comparative analysis among the whole-genome sequences of *G. hirsutum*, *G. arboretum*, and *G. raimondii* revealed that polyploidization events occurred both before and after the AADD lineage emerged and led to the expansion of the cotton genome [32,34]. In the present study, we have identified 320 *ACO* genes belonging to tetraploid cotton species *G. hirsutum* while 187 and 181 *ACO* genes were identified in each diploid cotton species, *G. arboreum* and *G. raimondii*, respectively. Therefore, compared with previous studies (11 *ACO* genes in pear and 9 *ACO* genes in melon) [42], cotton has a very large number of *ACO* members. Further analysis showed that a huge number of whole-genome duplication events (WGD) and tandem duplication events (TD) were found in cotton *ACO* genes. On the other hand, we found that a total of 227 *ACO* genes in the *G. hirsutum* genome have collinear genes in *G. arboretum* and *G. raimondii*. These results suggested that WGD and TD events play a leading role in the large-scale expansion of *ACO* genes in diploid cotton species, and they conserved well in allopolyploidization events. Selection pressure analyses further revealed the evolution process of the *ACO* genes in cotton. The Ka/Ks analysis showed that the Ka/Ks values between *Ga* and the At subgenome of *Gh*, Gr and the Dt subgenome of *Gh*, and the At and Dt subgenomes of *Gh* were larger than the value between *Ga* and *Gr*, indicating more positive selection after allopolyploidization than before allopolyploidization in cotton. Altogether, we surmised that the main reason for the existence of so many *ACO* genes in cotton is probably gene duplication events and that positive selection after allopolyploidization further drove the development of diverse functions and the retention of *ACO* genes in cotton.

### 3.2. GhACO106_At Probably Regulates Cotton Flowering Time

Promoters contain diverse varieties of important cis-elements that can accurately bind to template DNA to stimulate or inhibit gene transcription. Therefore, the functions of genes can be inferred by cis-element identification. In this investigation, the promoter analysis of *GhACOs* revealed that the cis-elements of *GhACOs* can respond to stress, phytohormones, light, and circadian factors, indicating the possible involvement of *GhACOs* in these processes (Figure 1). For *GhACO106_At*, it also contained the cis-regulatory element that responds to light, which can affect the growth periods of crops. The expression of *GhACO106_At* shows clear differences in expression in early- (Yanzao2) and late-maturing cotton (STS458; Figure 5a). These results further confirmed the function of this gene in early- and late-maturing varieties. Because flowering time is an important indicator of early and late maturity, we predicted that *GhACO106_At* may regulate flowering time. To further investigate the function of *GhACO106_At*, transgenic *Arabidopsis* lines overexpressing *GhACO106_At* were obtained, and it was discovered that all three transgenic lines had an early flowering phenotype (Figure 5b,c). Furthermore, previous studies have confirmed that ethylene can regulate vegetative growth and flower formation in plants [43,44,45,46,47]. For example, ethylene can enhance photosynthetic performance, promote vegetative growth, and increase the number of soybean flowers [47]. Therefore, based on the gene expression results and the phenotype results obtained in transgenic *Arabidopsis* as well as reports on the regulatory functions of ethylene in plant growth and development, it can be inferred that *GhACO106_At* probably functions in the regulation of cotton flowering time by controlling the synthesis of ethylene.

### 3.3. GhACO106_At Regulates the Growth of Roots and Salt Stress Resistance

Studies have reported that salt stress has a significant inhibitory effect on the germination of seeds. For cabbage, the germination and germination rate were significantly affected by different concentrations of salt stress [48]. In previous studies on sunflower and *Arabidopsis*, it was reported that ethylene can stimulate seed germination by inhibiting the effect of ABA [49,50]. In *Stylosanthes humilis*, within a given pH condition, the energy metabolism and embryo growth potential of seeds under salt stress may be regulated by the flexibility provided by the biosynthesis of ABA and ethylene [51]. Furthermore, in *Arabidopsis*, Yu et al. found that the nucleoplasm distribution of COP1 was antagonistically regulated by salt stress and ethylene, and then resulted in the controlling of seed germination via the COP1-mediated down-regulation of HY5 and ABI5 [52]. In this study, under the normal medium without salt stress, the germination rate of *GhACO106_At* transgenic *Arabidopsis* and the wild type is almost the same. However, with the increment of salt concentration, the germination rate of the wild-type *Arabidopsis* was significantly lower than that of the wild type, indicating that *GhACO106_At* could promote the germination under salt stress. Likewise, the change in seed germination in our results, we speculate, was also mediated by ethylene, and the biosynthesis of ethylene was regulated by *GhACO106_At.*

As roots are the functional organ responsible for nutrient acquisition, soil anchorage, and environmental interactions, their proper growth and development are critical for plants. Ethylene has been reported to play an important role in the growth and development of *Arabidopsis* roots, including the elongation of the main roots and the formation of lateral roots. In *Arabidopsis*, studies found that the ethylene-insensitive *Arabidopsis* mutant *eto1-1* exhibits a shorter primary root length; the enhancement of ethylene synthesis could promote the lateral root primordia’s initiation; and the coordinated complex consisting of EIN3/EIL1 and RHD6/RSL1, which is activated by ethylene, can promote root hair growth [53,54,55]. Besides, salinity is the most important environmental factor that inhibits root water uptake and slows the growth of plants. Studies have shown that ethylene acts as a signal of salt stress [56]. In *Arabidopsis*, salt stress increased the detectable levels of emanated ethylene as well as the expression of *ACS* genes encoding *ACS2* and *ACS7* transcripts [57]. In cotton, the expression of multiple ACOs is also induced by salt stress [58]. As for *GhACO106_At* in this research, compared to WT, the L2 seedlings exhibited significantly longer primary roots and much more lateral roots. This result suggested a positive role of *GhACO106_At* in plant root development, and this role is possibly implemented by regulating the biosynthesis of ethylene. Moreover, with the increase in salt concentration, the proline content of L2 increased more significantly than that of WT, and the content of MDA was significantly lower than that of WT in the medium of 100 and 150 mmol·L^−1^ NaCl. These results further demonstrated that overexpression of *GhACO106_At* can significantly enhance the resistance of plants to salt stress.

Altogether, our results revealed that the overexpression of *GhACO106_At* can promote seed germination and improve plant root development under normal and salt stress environments, as illustrated by higher germination rates, stronger root growth, and less damage degree of plants. In view of previous studies about the function of ethylene in seed germination, root growth, and salt stress, we speculate that the realization of all functions of GhACO106_At in this study may also be mediated by its regulation of ethylene biosynthesis.

## 4. Materials and Methods

### 4.1. Identification and Phylogenetic Analysis of ACO Genes in Cotton

The ZJU.V2, WHU and JGI versions of the protein databases of *G. hirsutum*, *G. arboretum*, and *G. raimondii*, respectively, were downloaded from the CottonGen website (https://www.cottongen.org/data/download, accessed on 12 May 2020) [59]. The two relevant domain files, DIOX_Nhmm (PF14226) and 2OG-FeII_Oxy.hmm (PF03171), were downloaded from the Pfam website (http://pfam.xfam.org/, accessed on 12 May 2020) [60] and used as queries to search against the three *Gossypium* species protein databases using HMMER3 (http://hmmer.org/, accessed on 15 May 2020) with an *e*-value threshold of 1 × 10^−5^. The Hmmsearch hits were submitted to the Pfam website to confirm those putative ACOs that contained the two target domains. The protein sequences of the ACOs were aligned using Clustal Omega with the default parameters (https://www.ebi.ac.uk/Tools/msa/clustalo/, accessed on 22 May 2020). The resulting alignments were used as the input file for MrBayes v3.2.5 to construct the corresponding phylogenetic tree with the following settings: the evolutionary model set to the GTR substitution model, gamma-distributed rate variation across sites, Ngen = 1,000,000, and Samplefreq = 100 [61]. The theoretical Mw, pI, and GRAVY values of the identified *ACOs* in this research were calculated at the ExPASy website (http://web.expasy.org/protparam/, accessed on 26 May 2020) [62]. The lengths and positions of the DIOX_N and 2OG-FeII_Oxy domains of each *ACO* gene were predicted using PfamScan (https://www.ebi.ac.uk/Tools/pfa/pfamscan/, accessed on 2 June 2020) and displayed in the phylogenetic tree using iTOL v4 (https://itol.embl.de/, accessed on 5 June 2020) [63]. The conserved motifs in ACOs were identified with MEME v5.0.5 (http://meme-suite.org/tools/meme, accessed on 8 June 2020) with the following parameters: site distribution, 0 or 1 occurrence per sequence; number of motifs, 10; and width between 6 and 50 [64].

### 4.2. Analysis of ACO Gene Duplication Event and Selection Pressure in Cotton

BlastP (*E* < 1 × 10^−10^, top five matches and m8 format output), Dupgen_finder (https://github.com/qiao-xin/DupGen_finder, accessed on 10 June 2020), and MCScanX (with default parameters) were employed to search for intragenomic and intergenomic gene duplications [65,66]. The protein sequences and coding sequences of each gene pair were aligned using MAFFT software and transformed into an AXT format via the pParaAT pipeline [67,68]. The Ka, Ks, and evolutionary constraint (Ka/Ks) values between each gene pair were calculated with Kaks_calculator (v2.0) using the method of Nei and Gojobori [69,70].

### 4.3. Expression and Co-Expression Analysis of GhACOs

Online available transcriptome data on tissue-specific and abiotic stress-related expression were downloaded from the NCBI SRA database (accession numbers PRJNA490626 and PRJNA532694). The RNA-seq data were analyzed based on the methods of Chen et al. [71]. Specifically, the raw RNA-seq reads were filtered using Trimmomatic with the default parameters, and clean RNA-seq reads were obtained. The clean RNA-seq reads were mapped to the reference genome using HISAT2, and SAM format data were obtained. The SAM format data were transformed into BAM format data by SAMtools. The BAM files were assembled into transcripts, and fragments per kilobase of transcript per million mapped reads (FPKM) values were generated using StringTie. Co-expression networks were constructed using the WGCNA package (v1.69) [72], and the networks were visualized with the help of Cytoscape (v3.7.2; http://www.cytoscape.org/, accessed on 5 March 2021).

### 4.4. Expression Analysis of GhACO106_At

The upland cotton varieties Yanzao2 and STS458 were planted in the experimental field of the Cotton Research Institute of the Chinese Academy of Agricultural Sciences and grown under routine field management methods. After the cotyledon flattened, the apical buds of Yanzao2 and STS458 were collected at the first, second, third, fourth, and fifth true leaf flattening stages. The TRIzol extraction reagent (Tiangen Biochemical Technology (Beijing) Co., Ltd.) was used to extract total RNA from the bud samples. A PrimeScript RT reagent kit with gDNA Eraser (Perfect Real Time, TaKaRa, China) was used to synthesize cDNA. Specific primers for *GhACO106_At* were designed with Oligo7 for real-time fluorescent quantitative RT-PCR. The forward primer was 5’-TGAAAGCGCTGGTAGATTCGGG-3’, and the reverse primer was 5’-TCATTGTTGGTGGGGCTTCCAG-3’. Each experiment was designed with three biological and three technical replicates. Quantitative RT-PCR was performed on an ABI 7500 Real-Time PCR system analyzer (Applied Biosystems) using UltraSYBR^®^ Mixture (Low ROX; CWBIO). The 20 μL reaction volume contained the following components: 10 μL of UltraSYBR Mixture (Low ROX; 2×), 0.4 μL of the PCR forward primer (10 μM), 0.4 μL of the PCR reverse primer (10 μM), 0.8 μL of cDNA, and 8.4 μL of ddH_2_O. The amplification was performed using the following protocol: a pre-denaturation step at 95 °C for 10 min; 40 cycles of (95 °C for 10 s, 60 °C for 30 s, and 72 °C for 32 s); a melting curve step at 95 °C for 15 s, 60 °C for 1 min, 95 °C for 15 s, and 60 °C for 15 s. The cotton endogenous *histone-3* (NCBI gene ID: 107950429) and *Arabidopsis* endogenous *Actin 2* (NCBI gene ID: 821411) were used as the internal standard to normalize the relative expression levels of *GhACO106_At* in cotton and *Arabidopsis,* respectively. Each experiment was designed for three biological replicates. The results of fluorescence quantitative dissolution curve analysis are shown in Supplementary Figure S4.

### 4.5. Screening of GhACO106_At overexpressing Arabidopsis

The open reading fragment (ORF) of *GhACO106_At* was inserted into a 35S promoter-driven vector (pBI121) to construct the 35S:*GhACO106_At* vector. This vector was then transformed into the *Agrobacterium* LBA4404 strain, which in turn was used to transform *Arabidopsis* via the floral-dip method [73]. The screening and verification of positive plants from T_0_ generation seeds to T_3_ generation seeds were performed based on the methods of Gu et al. [74].

### 4.6. Salt Stress Treatments

WT and *GhACO106_At*-overexpressing *Arabidopsis* seeds were planted in plates with 1/2 MS media and different salt treatments (50 mmol·L^−1^, 100 mmol·L^−1^, 150 mmol·L^−1^, and 200 mmol·L^−1^ NaCl). The plates were stratified at 4 °C for 2 days in darkness and then kept in a growth chamber under controlled conditions (22 °C, 70% relative humidity, and a 16/8 h light/dark cycle). On the 8th day after the seeds were planted, their germination and root length were recorded.

### 4.7. Statistical Analyses

The 2^−ΔΔCT^ method was used to analyze the experimental results to calculate relative expression of *GhACO106_At* at the first, second, third, fourth, and fifth true leaf flattening stages of Yanzao2 and STS458 [75]. Root length determination was independently performed on WT and L2 plants in 3 media, each of which contained 10 seedlings. Germination was recorded when the embryo broke through the seed coat, and the standard for germination was considered a germinated embryo length exceeding half of the seed length. The percentage of the total germination rate was determined as the ratio of the number of germinated seeds (n) to the number of tested seeds (N) multiplied by 100 (100 × n/N). The determination of proline and MDA contents was performed based on the Beijing Solebold Biochemical Kit manual and was repeated three times. The degree of the differences between transgenic and WT *Arabidopsis* under the same salt concentration was evaluated with independent samples t-tests. Microsoft Excel was used to perform the *t*-tests (significant difference, *p* < 0.05; very significant difference, *p* < 0.01).

## 5. Conclusions

In the current study, cotton *ACO* genes were identified from *G. hirsutum*, *G. arboretum*, and *G. raimondii*. In total, 332 *GhACOs*, 187 *GaACOs*, and 181 *GrACOs* were identified, and their physicochemical properties, domain architectures, phylogenetic relationships, syntenic relationships, and spatiotemporal expression were analyzed. These *ACO* genes were divided into four subgroups based on phylogenetic analysis. The upstream analysis of *GhACOs* revealed that elements in the promoters of these *ACO* genes respond to stress, phytohormones, light, and circadian factors, indicating the possible involvement of *GhACOs* in these processes. Gene duplication analysis showed that WGD and tandem duplication events were the major forces driving the generation of *ACO* genes. The expression and co-expression analyses illustrated that most *GhACOs* were not only widely expressed in various tissues but were also coexpressed with other genes in response to salt and drought stress. Furthermore, the functional investigation of *GhACO106_At* showed a significantly reduced flowering time and an increased salt resistance in *GhACO106_At* transgenic *Arabidopsis*, suggesting that *GhACO106_At* probably participates in the regulation of flowering time and the response to salt stress.

## Figures and Tables

**Table 1:** Rosette leaf number at bolting in transgenic and WT *Arabidopsis*.

Line	Number of Plants	Flowering Time (Days after Sowing)	Number of Rosette Leaves (Mean ± SD)
1	32	27.0 ± 0.44 *	9.75 ± 0.87 ***
2	30	27.7 ± 0.66 *	10.55 ± 0.84 ***
3	28	27.3 ± 0.53 *	10.04 ± 0.84 ***
WT	30	32.8 ± 0.47	11.93 ± 0.80

1–3: Transgenic lines. WT: wild type. Student’s *t*-test: * *p* < 0.05; *** *p* < 0.001.

## Data Availability

All the genome sequences analyzed in this project were retrieved from Cottongen (https://www.cottongen.org/ (accessed on 17 August 2021)), the JGI database (https://www.phytozome.net (accessed on 17 August 2021)), and the National Center for Biotechnology Information (NCBI) database (https://www.ncbi.nlm.nih.gov/ (accessed on 17 August 2021)).

## Supplementary Materials

The following supplementary materials are available online at https://www.mdpi.com/article/10.3390/plants10081699/s1: Figure S1: Phylogenetic relationships and subfamily designations of ACO proteins from *G. hirsutum*, *G. arboreum*, and *G. raimondii*; Figure S2: Conserved motifs in cotton ACO proteins; Figure S3: Expression characteristics in different tissues of *GhACOs*; Figure S4: The results of RT-PCR fluorescence quantitative dissolution curve analysis in cotton and *Arabidopsis* of *GhACO106_At*; Table S1: Information on *ACO* genes in cotton; Table S2: Details of conserved motifs in cotton ACO proteins; Table S3: Information on cis-elements identified in the promoters of *GhACOs*; Table S4: Duplication events of *ACO* genes in cotton species; Table S5: Expression patterns of *GhACOs* in different cotton tissues; Table S6: Expression characteristics of *GhACOs* under various stresses; Table S7: List of genes coexpressed with *GhACOs*.
