# Supplementary material for: Genome-Wide Identification and Functional Investigation of 1-Aminocyclopropane-1-carboxylic Acid Oxidase (ACO) Genes in Cotton
Source: Plants (Basel). 2021 Aug 18;10(8):1699. doi: 10.3390/plants10081699 (PMC8402218; doi:10.3390/plants10081699)
Supplement: Supplementary file 1 [file plants-10-01699-s001.zip › Supplementary files/Supplementary Figure S2.pdf]

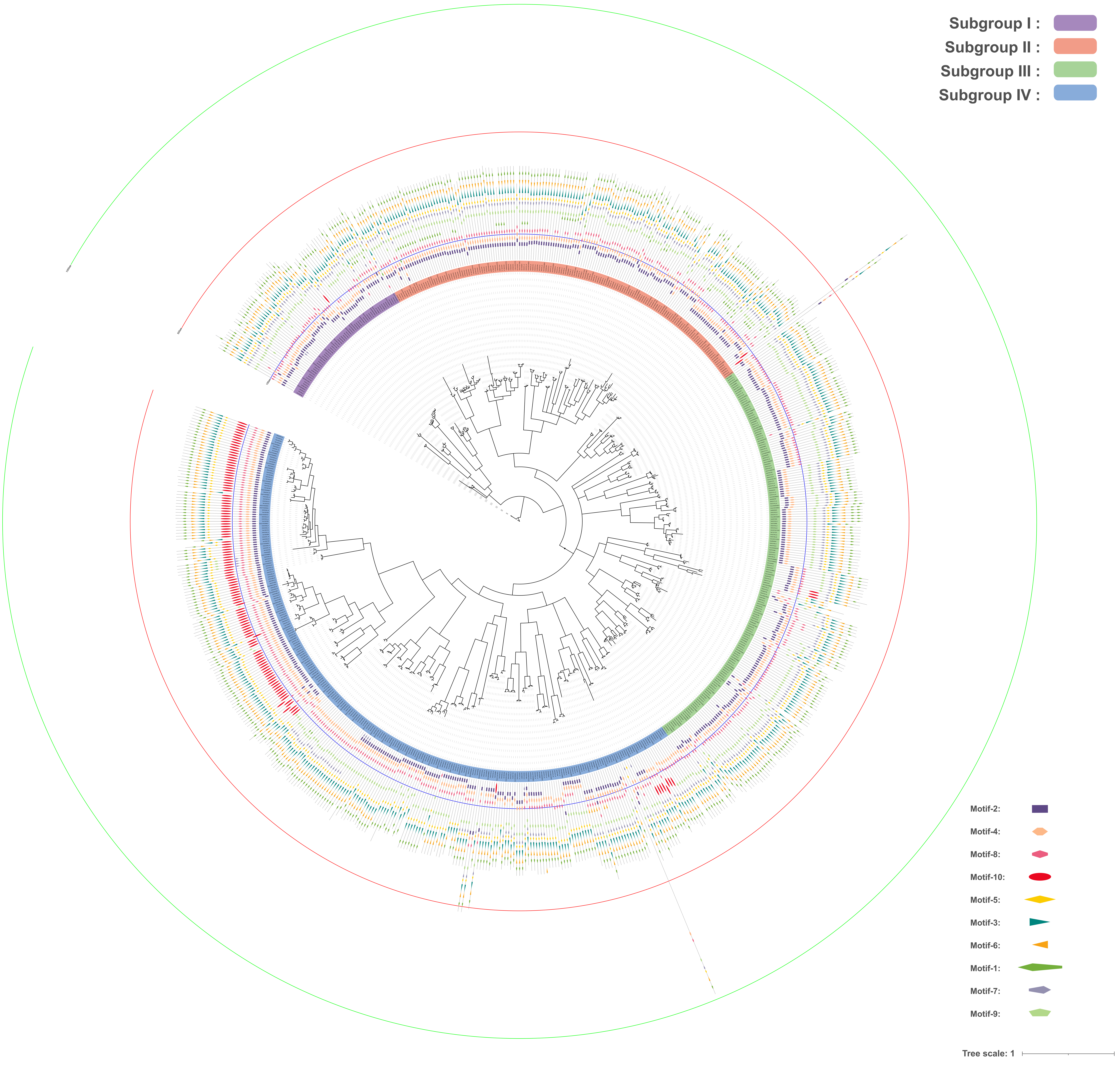

Subgroup I :
Subgroup II :
Subgroup III :
Subgroup IV :
Motif-2:
Motif-4:
Motif-8:
Motif-10:
Motif-5:
Motif-3:
Motif-6:
Motif-1:
Motif-7:
Motif-9:
Tree scale: 1
