# Supplementary figures and images for: Genome-Wide Identification and Functional Investigation of 1-Aminocyclopropane-1-carboxylic Acid Oxidase (ACO) Genes in Cotton
Source: Plants (Basel). 2021 Aug 18;10(8):1699. doi: 10.3390/plants10081699 (PMC8402218; doi:10.3390/plants10081699)

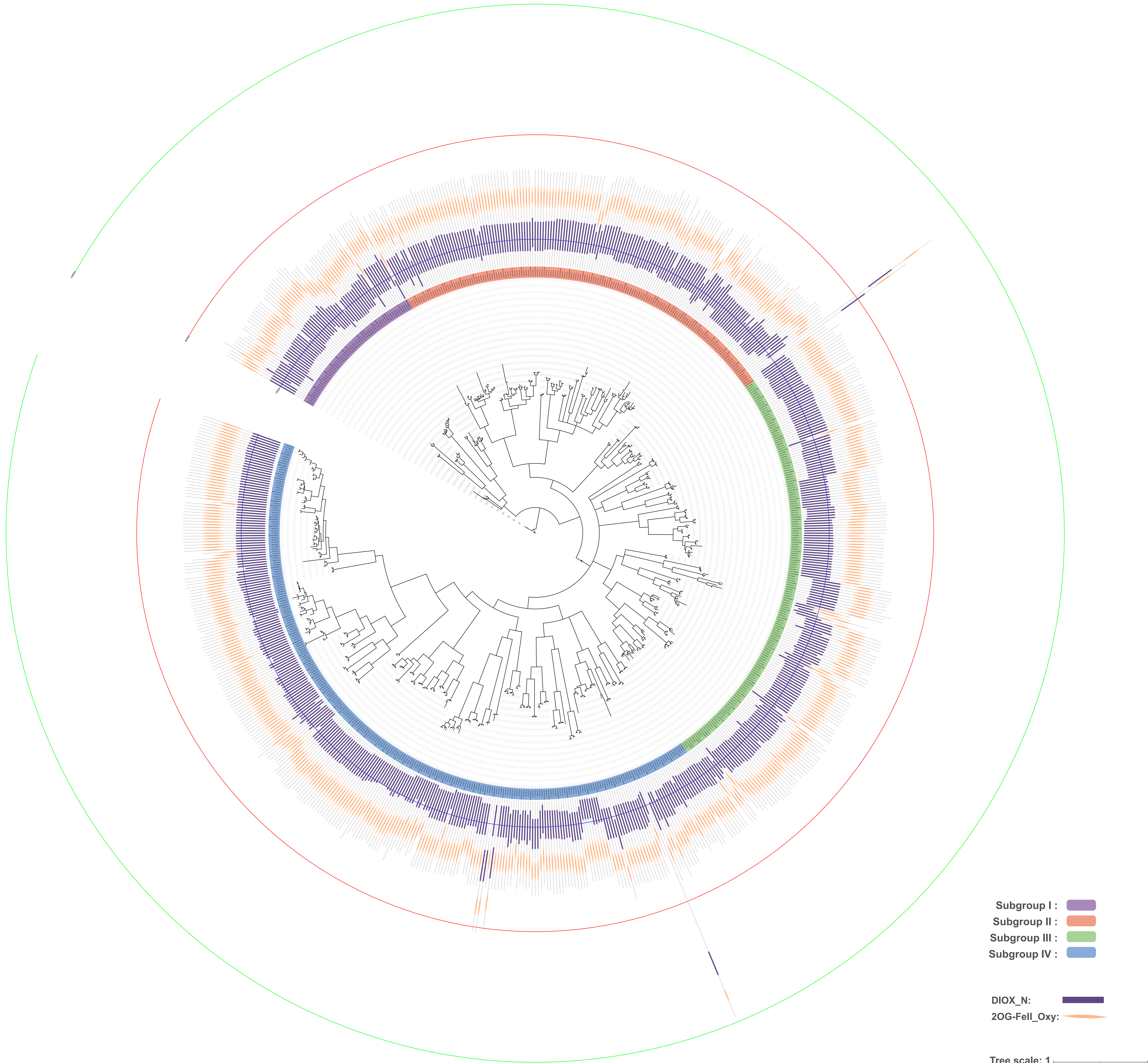

Supplement: Supplementary file 1 [file plants-10-01699-s001.zip › Supplementary files/Supplementary Figure S1.pdf]

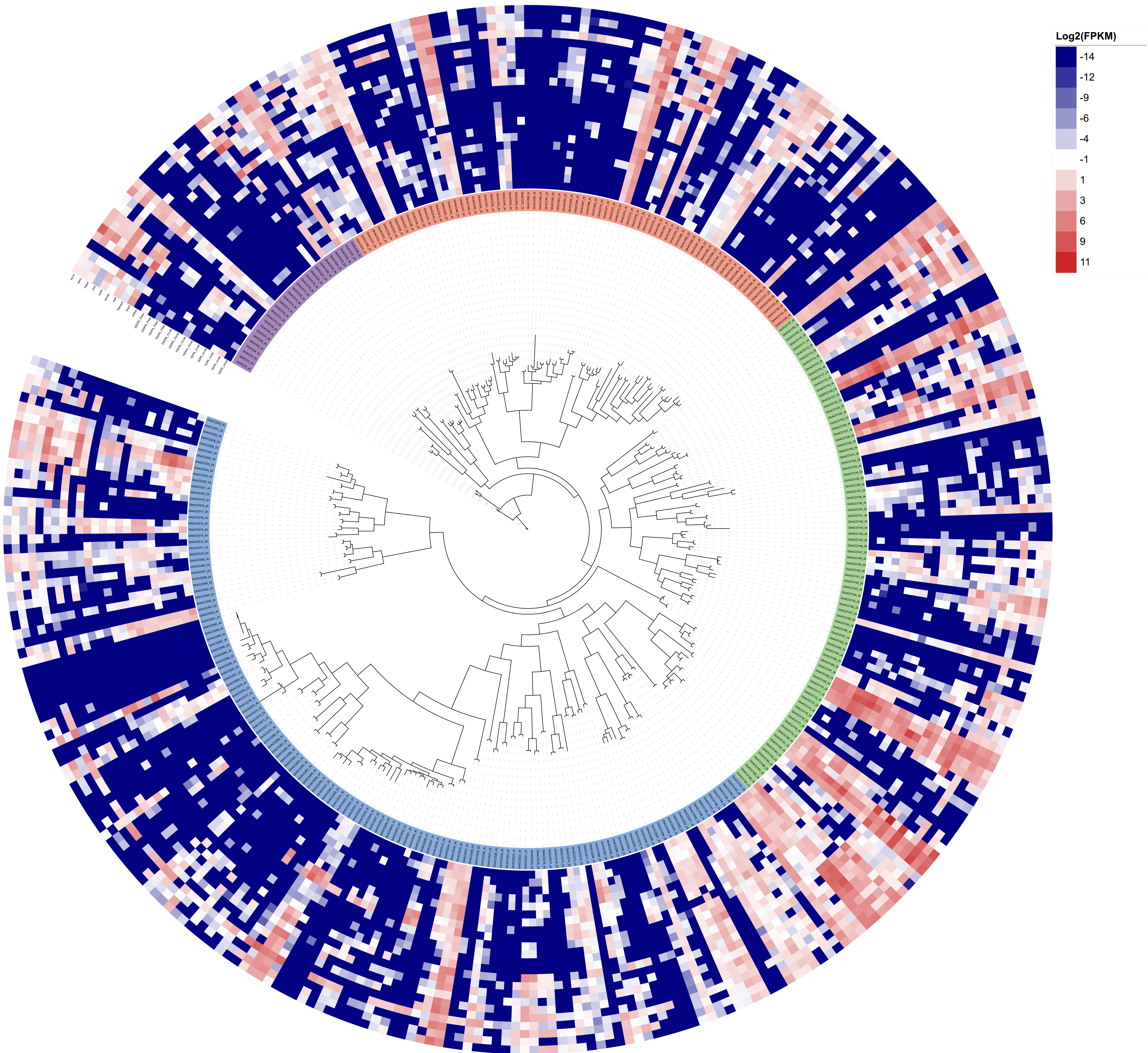

Supplement: Supplementary file 1 [file plants-10-01699-s001.zip › Supplementary files/Supplementary Figure S3.pdf]

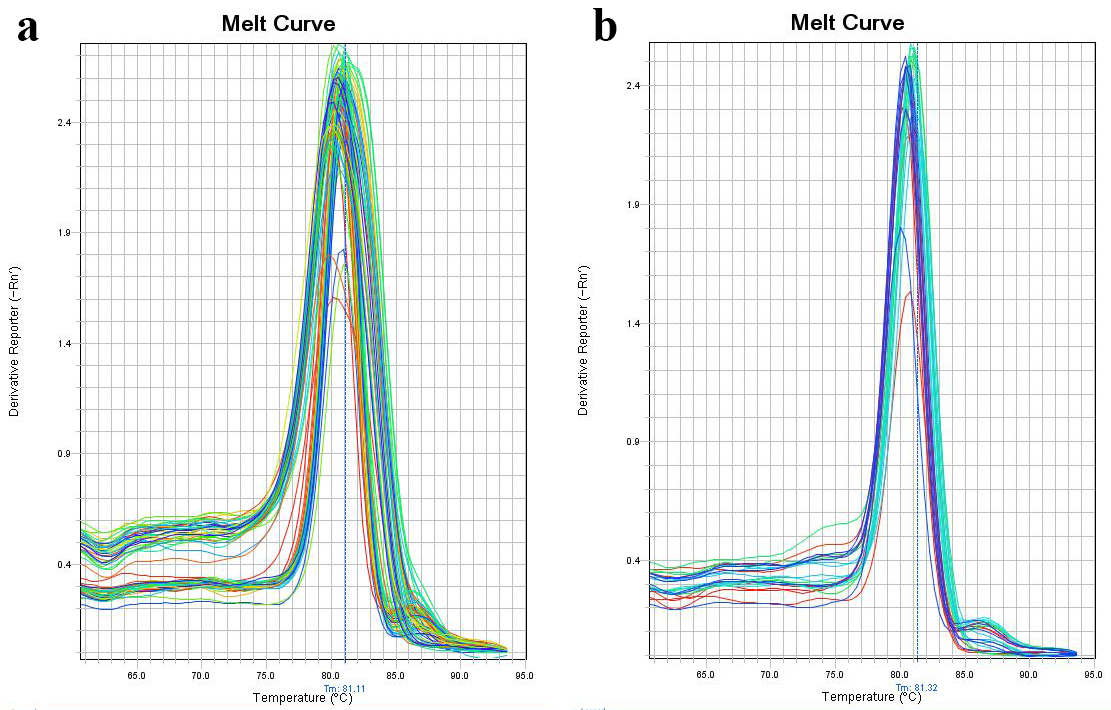

Supplement: Supplementary file 1 [file plants-10-01699-s001.zip › Supplementary files/Supplementary Figure S4.tif]
